# A novel environmental restoration method for an abandoned limestone quarry with a deep open pit and steep palisades: a case study

**DOI:** 10.1098/rsos.180365

**Published:** 2018-05-30

**Authors:** Hanxun Wang, Bin Zhang, Xueliang Bai, Lei Shi

**Affiliations:** 1 School of Engineering and Technology, China University of Geosciences (Beijing), Beijing, 100083, People's Republic of China; 2 Key Laboratory on Deep Geodrilling Technology, Ministry of Land and Resources, Beijing, 100083, People's Republic of China

**Keywords:** abandoned limestone quarry, field geological survey, environmental restoration, artificial filling slope, revegetation

## Abstract

In general, exploitation of rock materials, such as limestone or granite exploitation, can cause serious damage to the environment near a mine area. With economic development and the ever-increasing demand for ore resources, mining activities have induced very serious environmental issues in China. Therefore, environmental restoration work around mines in China is urgently required. This study explores the Chuankou open-pit limestone quarry in Tongchuan City, Shaanxi Province, Northwest China, as the engineering case. The environmental issues caused by over 40 years of limestone exploitation, including land degradation, land occupation, dust pollution and potential geological disasters, were investigated. Combining the characteristics of this quarry with a summary of previous studies on environmental restoration work, this paper proposes a novel and systematic method that was comprehensively carried out through engineering and revegetation measures. The engineering measure, that is, the construction of an artificial slope by using local abandoned construction materials, solved the environmental problems in this quarry and provided site conditions favourable for revegetation. The revegetation measure restored the local ecosystem. This method provides both a new idea for the sustainable development of a mining area and a useful reference for analogous engineering cases.

## 1. Introduction

Mining provides important raw materials for national economic development and the progress of human civilization and is a mainstay industry of many countries and regions [1]. Moreover, open-pit mining is generally preferred in mining operations due to its applicability in all operable areas of exposed rock, minimum production loss and high production rate [2], especially for ores such as limestone, granite and iron ore that are exposed at the surface. Innumerable open-pit mines have been built around the world, such as the Kalgoorlie Superpit open-pit gold mine in Western Australia [3], the Chilean Chuquicamata open-pit copper mine [4] and the Bingham Canyon Copper Mine [5,6]. Indeed, in these mines, high-quality minerals have been produced, contributing to social development; however, the local environments have been completely destroyed. In addition, during open-pit mining activities, 2–11 times more land is degraded than that by underground mining [7].

Therefore, people and governments are paying an increasing amount of attention to the protection of mining environments, especially the recovery of abandoned open-pit mining environments. Western developed countries have a long history of mining, and many mines have been abandoned. Since the 1920s, environmental restoration of mines, especially open-pit mining land, has been carried out, and relevant laws have been promulgated to recover the mining environments [8] in Canada, Germany and Australia [1,9]. The environmental restoration of a quarry area in northeastern Spain [10], the treatment engineering of the Berkeley Pit in the USA [11] and other projects have provided valuable experience for environmental restoration of mines. Restoration in China began in the late 1950s and early 1960s. Although this work started relatively late, a great deal of attention and investment is still devoted to mine environmental management. In recent years, many environmental management projects have been carried out. After decades of environment treatment, the land reclamation rate of the Malan iron mine in Hebei reached 85%; that of the Pingguo aluminium mine in Guangxi reached 73%; that of the Yongping copper mine in Jiangxi reached 55%; and that of the Ankang gold mine in Shanxi reached 69% [1]. However, many issues remain, and the environmental restoration work should be continued.

In recent years, with the development of the economy in China, non-metallic materials such as limestone are widely used as building materials, and significant amounts of industrial raw materials are exploited. During 2010–2016, the annual average consumption of limestone used to produce cement in China exceeded 2.5 billion tons [12,13]. As a result, limestone exploitation caused a strong adverse impact on integrated ecological systems near these mining areas [14–17]. Considering the typical characteristics of limestone exploitation, the environmental damages caused by limestone exploitation mainly include species extinction, wetland destruction [18–21], dust and particulate matter pollution [22,23], and potential mining geological hazards [24] etc. In addition, these limestone exploitation activities usually also have a significant adverse effect on hydrological processes [25–27], consuming significant quantities of water [28,29] and contaminating the surface water and shallow groundwater [30]. Many studies have examined how to treat environmental problems and restore the ecosystems of abandoned limestone quarries. Robert *et al*. [31] discussed the effect of spontaneous succession as a vegetation restoration tool and agreed that it is effective. Gilardelli *et al*. [32] studied the vegetation restoration effect of different methods and provided valuable suggestions for limestone quarry environmental restoration. In general, if suitable environmental conditions occur, natural succession will have a good recovery effect [33–35]. However, for a limestone quarry, restoring the ecosystem is lengthy and difficult work, because the vegetation and layers of soil are often completely removed during resource extraction [36]. Therefore, appropriate human intervention is necessary [37]. Considering these adverse factors, this paper takes the Chuankou limestone quarry with a deep open pit and steep palisades as an engineering case. Moreover, combining the characteristics of this quarry with a summary of previous studies on environmental restoration work, a novel method was proposed, which involves comprehensive and multidisciplinary measures and could restore the local environment and ecosystem. The idea was comprehensively carried out by implementing and integrating complex engineering and revegetation measures. Through the engineering measure, an artificial slope was filled with local abandoned building materials that included rock debris, construction waste and loess; this measure solved the environmental problems induced by the limestone exploitation activities over 40 years, including land degradation, land occupation, dust pollution and potential geological disasters. In addition, the slope provides site conditions favourable for artificial revegetation. Furthermore, the revegetation measure restored the local ecosystem. This study not only introduces a novel and effective idea to improve the sustainable development of an abandoned limestone quarry, but also provides a useful reference for analogous engineering cases.

## 2. Material and methods

### 2.1. Study area

**Figure 1.**
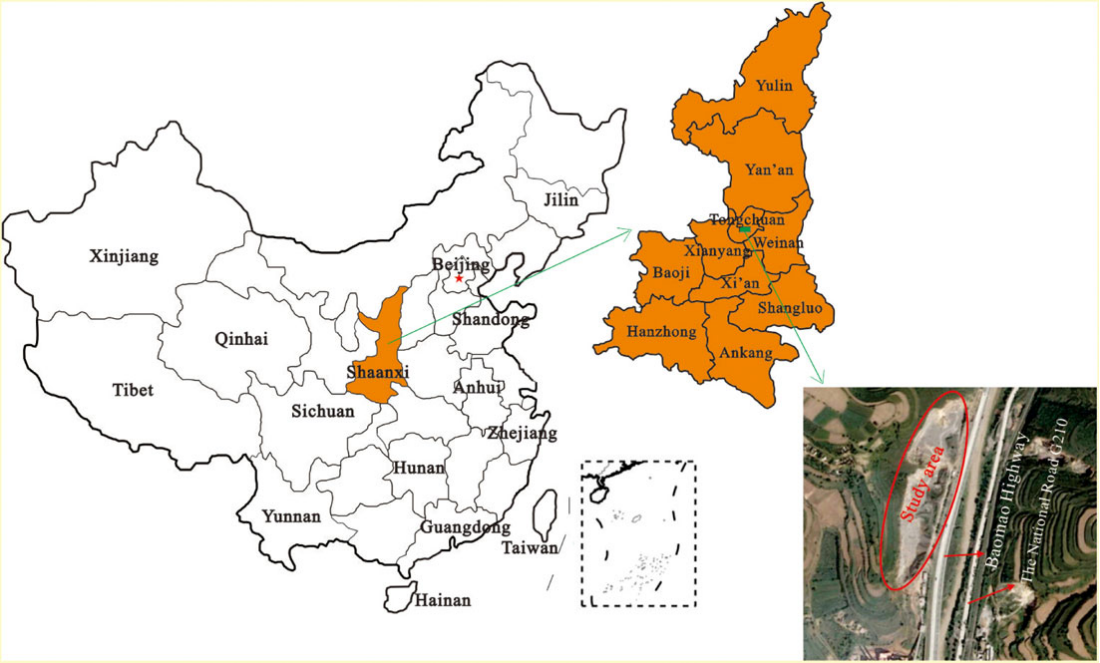
Geographical location of the mining area.

The quarry is located in Wangyi District, Tongchuan City, Shaanxi Province, Northwest China (figure 1), in the transition zone of the loess plateau of northern Shaanxi and the Guanzhuang Basin. The landform in the study area is loess hilly land. Because of fluviraption, the loess plateau-gully-slope morphological pattern is very well developed.

**Figure 2.**
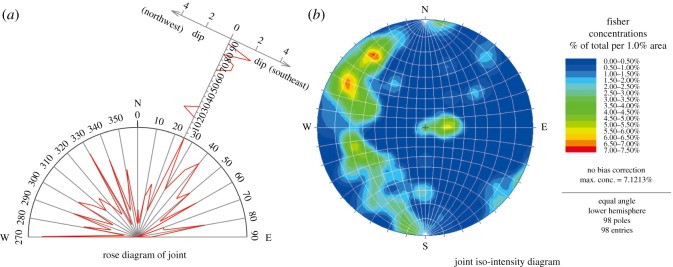
(*a*,*b*) Statistical figure of joints.

The geological tectonic activity in the study area is strong. Five normal faults have developed in the study area. Their trends are mainly in the east–west, northeast and northwest directions, and their dip angles are approximately 45°–70°. The joints in the limestone rock mass are extremely well developed (figure 2) [38]. The most preponderant joint trend is approximately 25°, and the most preponderant joint dip angle is approximately 85°. Therefore, the rock mass was severely fractured and fragmented.

The main lithology in the study area is the early Palaeozoic Ordovician Majiagou Formation (O_2_), which is a dolomitic limestone with a total thickness greater than 327 m, in which argillaceous interlayers developed. The limestone is off-white and has a microcrystalline structure. The limestone layer is nearly horizontal, with a strike of approximately 240°–280° and a dip angle of 15°–25°. The surface coverage is a Quaternary loess layer (Q_2–3_) and an artificial soil layer. The loess is sandy brown and contains caliche nodules. Moreover, the loess layer is widely distributed across the whole study area and has a thickness of approximately 46 m. The artificial soil layer consists of abandoned silty clay, gravel, limestone rock debris and construction waste, and is very loose.

The climate is a dry continental climate. The annual average temperature is 12.3°C. January is the coldest month and has an average temperature of −16°C, while July is the hottest month and has an average temperature of 24.8°C. Rainfall is low and concentrated, and the evaporation effect is strong. The average annual rainfall is 581 mm. In addition, the rainfall is concentrated in July, August and September, during which time the total rainfall reaches 342 mm and accounts for 57% of the annual rainfall. The groundwater level is deep. The main surface water is the Qishui River, located to the east of the quarry, and its distance from the quarry is approximately 500 m. The watershed is on the west side of the quarry area, and the catchment area of the mine area reaches 5.6 × 10^4^ m^2^. The surface run-off caused by rainfall, especially heavy rain, has a considerable impact on the slope.

The vegetation in the study area is mainly sparse and only includes a small amount of economically important forest and shrubs, such as *Robinia pseudoacacia, Rhus typhina, Prunus cerasifera* and *Calocedrus macrolepis*.

In summary, limestone reserves in this region are abundant, and limestone is a superior raw material for the production of high-quality cement. In the mid-nineteenth century, Tongchuan became the production base of cement and other building products for Shaanxi and even across the northwest region in China. In 2005, quarries were forcibly closed due to national policy and depletion of resources in the area. Nevertheless, the local eco-environment has been destroyed and geological hazards with high risk have been induced by the limestone exploitation activities; therefore, these circumstances should be given considerable attention.

### 2.2. Current environmental problems in the mine area

**Figure 3.**
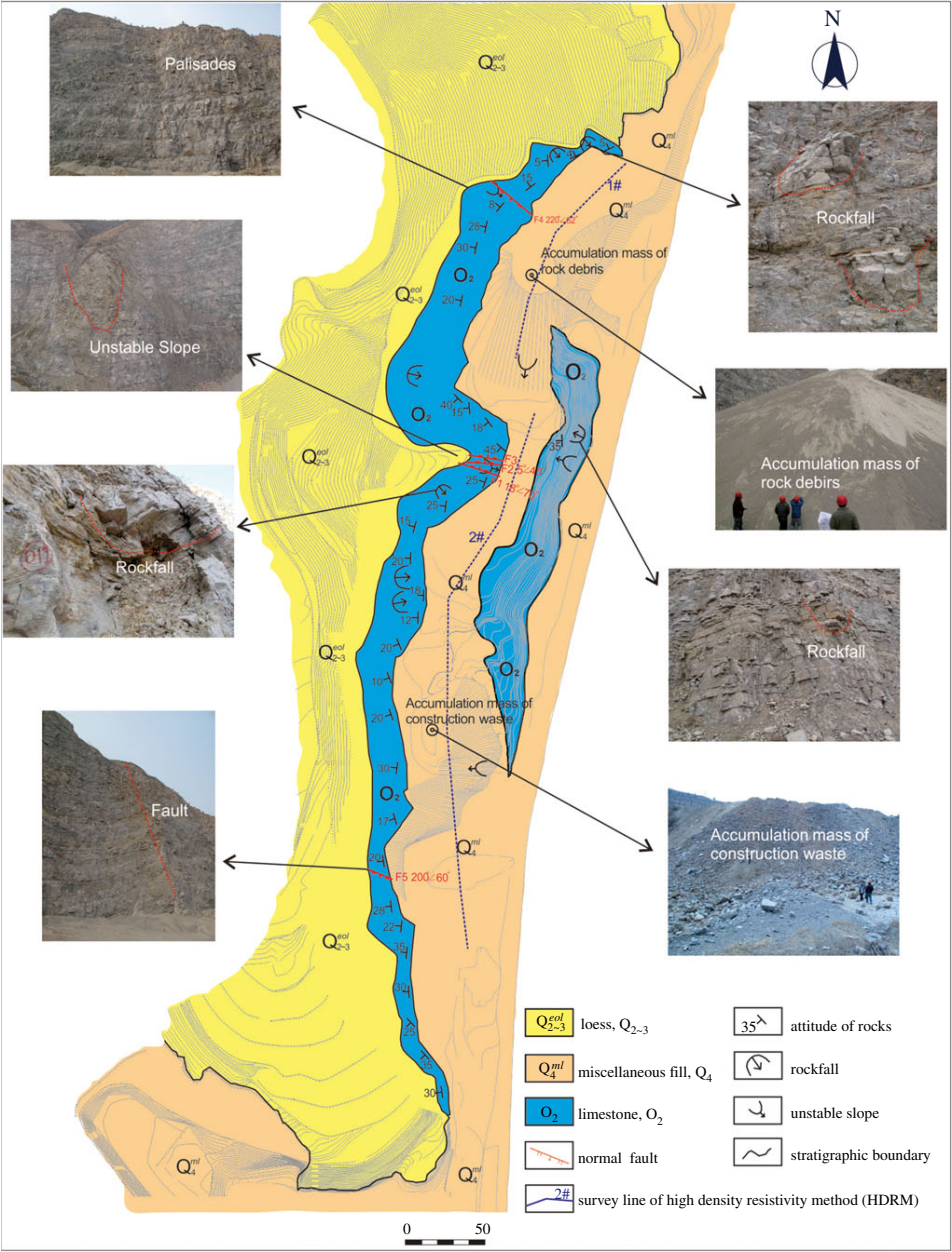
Geological environmental problems in the quarry area.

**Figure 4.**
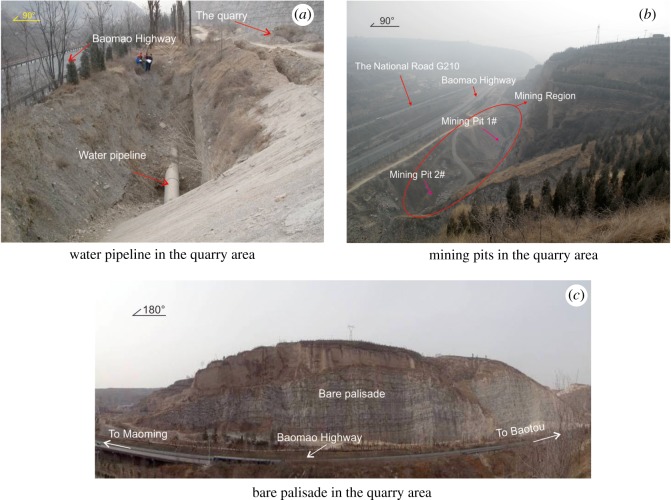
(*a*–*c*) Segmental environmental problems in the quarry area.

In this quarry, limestone exploitation activities over the last 40 years have destroyed the local environment and induced a series of environmental problems (figure 3). Moreover, due to the randomness of limestone mining and the accumulation of rock debris, many potential geological hazards have been generated, seriously threatening the safe operation of the traffic lifeline engineering projects to the east: the Baomao Highway, National Road G210 (figure 1) and the water pipeline located to the east of the pits (figure 4*a*).

#### 2.2.1. Land degradation

The original earth surface in the mine area has been completely stripped, which has destroyed the local ecosystem in the study area and surrounding region, and changed the original landscape and terrain. According to statistics, the area of the destroyed ecosystem region is approximately 0.5 km^2^ (figure 3). As a result, water loss and soil erosion have occurred at the surface, and an extensive amount of bedrock has been exposed (figure 4*c*); therefore, the establishment of vegetation is considerably limited. Moreover, two large-scale mining pits were created, in which the total capacity is more than 6.0 × 10^5^ m^3^ and the area is over 2.9 × 10^3^ m^2^ (table 1 and figure 4*b*), to the detriment of the surrounding environment.

**Table 1.** List of pit characteristics.

pit name	length (m)	width (m)	area (m^2^)	volume (m^3^)
1# pit	247	50–120	2.09 × 10^4^	4.45 × 10^5^
2# pit	168	10–75	8.39 × 10^3^	1.90 × 10^5^
total	—	—	2.93 × 10^4^	6.35 × 10^5^

#### 2.2.2. Land resource occupation and dust pollution

**Figure 5.**
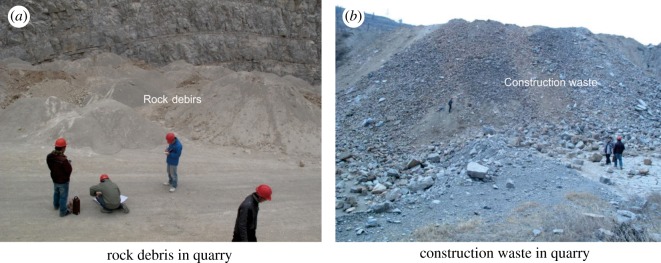
(*a*,*b*) Rock debris and construction waste accumulation in the quarry area.

**Figure 6.**
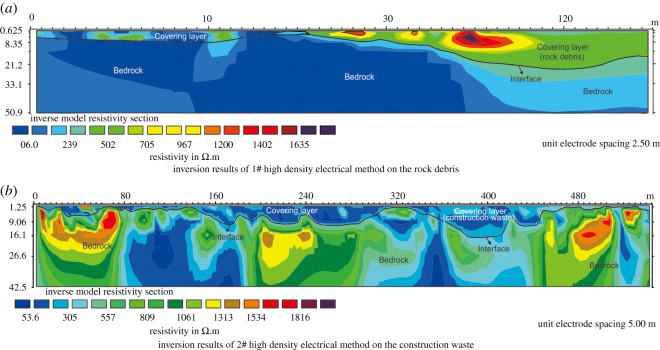
(*a*,*b*) Inversion results of the high-density electrical method.

A large amount of rock debris and construction waste was accumulated in the mine area (figure 3). To determine the thickness of the rock debris (figure 5*a*), construction waste (figure 5*b*) and covering layer in the study area, two test lines were designed along the bottom of the mining pits for high-density electrical measurement (figure 3). The 1# test line with a length of 180 m was used to reveal the thickness of the rock debris in the northern mine area. Figure 6*a* shows the inversion results for the 1# test line from the high-density electrical method and indicates that the maximum thickness of the accumulated rock debris was approximately 30 m. Combined with the field investigation, these results indicate that the rock debris occupied an area of 1.1 × 10^4^ m^2^ and a total volume of approximately 1.9 × 10^5^ m^3^. This debris mainly consisted of the remaining limestone debris, with its particle size in the range 0.1–0.5 cm. However, the 2# test line with a length of 500 m showed the distribution of the construction waste in the southern mine area and indicated that the maximum accumulation thickness of the construction waste was nearly 18 m (figure 6*b*). The construction waste occupied an area of approximately 1.5 × 10^3^ m^2^, and the total volume was approximately 8.0 × 10^3^ m^3^. The main particle size of the construction waste was in the range 5–20 cm, and the particle size of a part of construction waste blocks was even more than 50 cm.

The accumulated rock debris and construction waste was not compacted and was unconsolidated. These materials occupied and wasted the finite land resources and induced an enormous risk of dust pollution under the condition of windy weather and debris flows under the condition of heavy rain.

#### 2.2.3. Potential geological hazards

The bare palisade and loose accumulation mass caused a significant geological hazard risk. If these problems were not treated in a timely manner, rockfall, debris flow and other hazards would continually occur, endangering the traffic lifeline system to the east and other facilities. The potential geological hazards are as follows:

##### 2.2.3.1. Rockfalls

Through the field investigation, seven potential rockfall hazards were identified in the study area on the western high and steep rock slope of the mining pits (figure 3). Under the condition of heavy rain or other predisposing factors, these potential rockfall hazards could happen easily, threatening passing residents, workers or vehicles. During the survey, it was found that several small-scale rockfalls have occurred.

##### 2.2.3.2. Unstable slopes

There are five unstable slopes in the study area, including three unstable rock slopes and two loose accumulation slopes (figure 3), which represent a potential risk to the surrounding engineering facilities and the security of personnel.

##### 2.2.3.3. Debris flows

Loose accumulation of rock debris, construction waste and other materials in the covering layer could all provide an abundant source for potential debris flows. Considering the long, narrow terrain and the concentrated rainfall, the potential debris flows have a high likelihood of occurrence, greatly threatening the traffic lifeline system, water pipeline and nearby residents. According to the statistics, the potential debris flow area was approximately 7.8 × 10^3^ m^2^, and the volume of debris material was approximately 1.2 × 10^5^ m^3^.

## 3. Results

### 3.1. Environmental treatment

Because of the limestone exploitation activities, the local landscape and eco-environment have been destroyed, and a series of environmental problems, including land degradation, dust pollution, surface water loss and potential geological hazards, have been caused. Moreover, the loss of surface water and exposed bedrock make it very difficult for vegetation to spontaneously grow.

**Figure 7.**
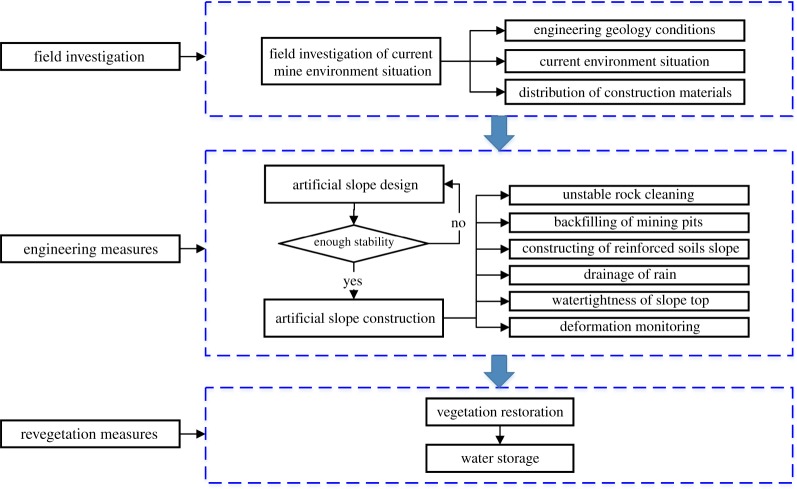
Flow chart of the mine geological environmental management and restoration measures.

Considering the characteristics of this quarry, a novel environmental restoration measure is proposed. The implementation processes are shown in figure 7. Through an engineering measure, this method takes full advantage of the local construction materials, including the rock debris, construction waste and abandoned loess. Using a mixture of these materials, an artificial slope is filled. The engineering measure eliminated the environmental problems induced by the limestone exploitation activities and provided site conditions favourable for artificial revegetation.

#### 3.1.1. Engineering measures

##### 3.1.1.1. Artificial slope design

**Figure 8.**
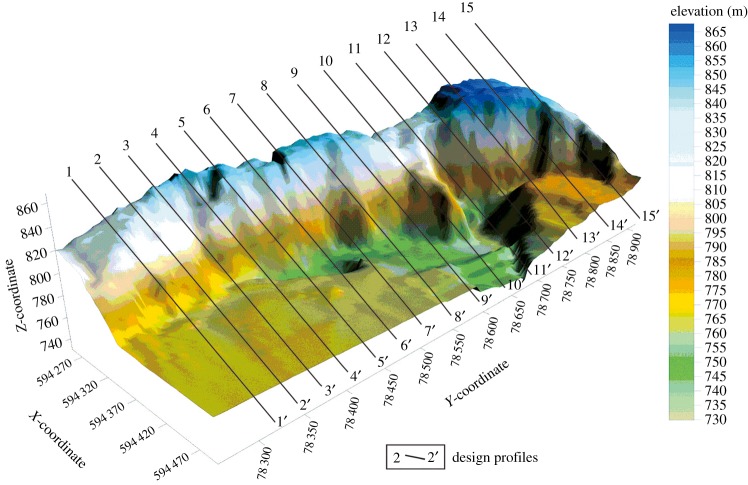
Current terrain and design of the artificial slope profiles.

Based on previous field investigation, an artificial slope was designed. In this case, according to the local terrain and conditions, the entire treatment region is divided into 15 profiles (figure 8). Then, each profile was designed and evaluated to guarantee the stability of the artificial slope according to the strength reduction finite-element method (FEM). The safety factor and sliding surface of each profile are listed in table 2. The safety factor of each profile of the artificial slope in the natural status is more than 1.88. Therefore the slope holds good stability.

**Table 2.** Stability analysis of each profile.

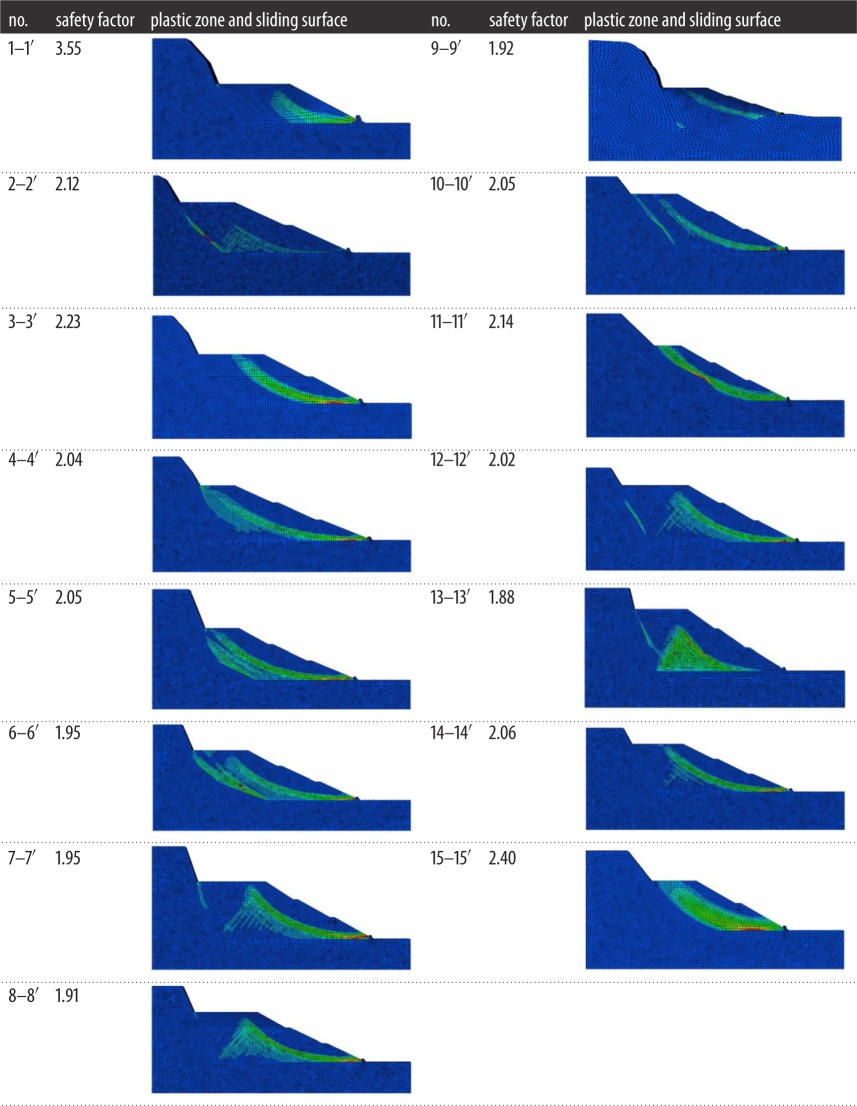

**Figure 9.**
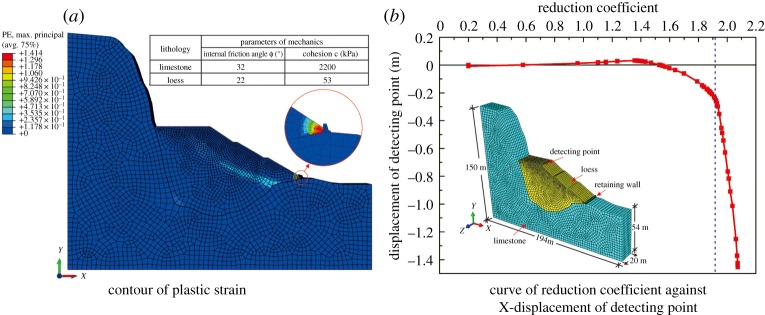
(*a*,*b*) Stability calculation results of the 9–9′ profile.

In addition, because the 9# profile has a steep pit and thick filling, it is selected as the typical example to study. Through laboratory tests, the physical and mechanical parameters of the loess and limestone are obtained (figure 9*a*). Based on the strength reduction FEM, the numerical model was built, and the slope stability was analysed quantitatively. The model range was 194 m in the *X* direction, 20 m in the *Y* direction and 150 m in the Z direction (figure 9*b*). The displacement boundary conditions were set as follows: both vertical and horizontal displacements were restrained at the bottom boundary, the horizontal displacement was fixed at the four lateral boundaries and the ground surface was free. The constitutive model is the Mohr–Coulomb model, and the load is the self-weight. Figure 9 shows the stability calculation results and indicates that the safety factor of the 9–9′ artificial slope is 1.92, which satisfies the stability requirement.

##### 3.1.1.2. Artificial slope construction

**Figure 10.**
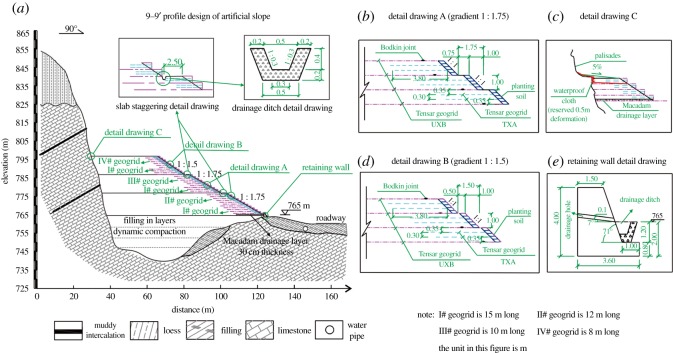
(*a*–*e*) Slope design of the 9–9′ profile.

The artificial slope is constructed with the following procedures:

*(1) Unstable rock cleaning*: the collapsible rocks located at the palisade should be cleaned up to ensure that the subsequent operation was safely implemented. The manual work and mechanical execution may be integrated.
*(2) Backfilling of the mining pits*: the mining pits were filled with rock debris, construction waste and loess (figure 10*a*). The materials should comply with the relevant technical standard. In this case, particles greater than 15 cm in diameter should be removed or broken. The pits were backfilled in layers. First, rock debris was filled and compacted to a 0.3 m thickness. The loess was placed over the previous filling layer and filled to a thickness of 0.3 m. This engineering procedure was repeated until the ground elevation reach the designed datum, which is a 765 m elevation in this case.
*(3) Constructing the reinforced soil slope*: above the datum, the artificial slopes were built using loess, and the slopes were enhanced with geogrid reinforcement (figure 10*a*). To improve the stability of the slope and facilitate subsequent construction, the slopes were set to some levels, and a platform and sidewalks were added to each level of the slope. The number of levels, the size of the platform and sidewalks, and the design of the slope surface should be determined according to the specific circumstances. The padding of the slope surface uses the loess that is suitable for the growth of plants to facilitate revegetation. In this case, the 9–9′ profile designs are shown in figure 10. To improve the stability of the artificial slope, a gravity retaining wall is built at the trailing edge of the first platform (figure 10*a*,*e*). The design and size of the retaining walls are shown in figure 10*e*.
*(4) Drainage of rain*: to prevent erosion on the slope surface due to rain, catchwaters were built in the cliff at a certain distance from the top of the cliff edge. Near the slope toe on every platform, drainage ditches were set (figure 10*a*,*e*). Along the contact line between the palisade and the artificial slope, discharge channels were constructed. At the bottom of the contact position between the gravity retaining wall and the slope, gravel drainage ditches were set with a certain thickness and width, and their slope angle depends on the local circumstances. In this case, the designs are shown in figure 10*e*.
*(5) Watertightness of the slope top*: to prevent rain flow in the slope mass, reducing the stability of slope, waterproof measures should be applied at the contact position between the palisade and the slope. In this case, composite geomembranes were made (figure 10*c*). One end of each of the composite geomembranes was pressed into the top position of the slope and was extended outwards for 0.5 m. The other end is fixed on the rock palisade over the slope top and hung approximately 2 m, and the middle part was wavy.
*(6) Deformation monitoring*: in general, the large subsidence deformation would be induced in the filling soil. Therefore, laying out deformation monitoring equipment is necessary. In this case, the instruments monitoring the subsident deformation were embedded in the soil surface of the slope, and those monitoring the horizontal deformation were installed in the interior of the slope; these instruments were used to monitor the subsidence and deformation for a period of time during the construction period and after the completion of the construction. Moreover, the stability of the artificial slope can be qualitatively analysed.

#### 3.1.2. Revegetation measure

**Figure 11.**
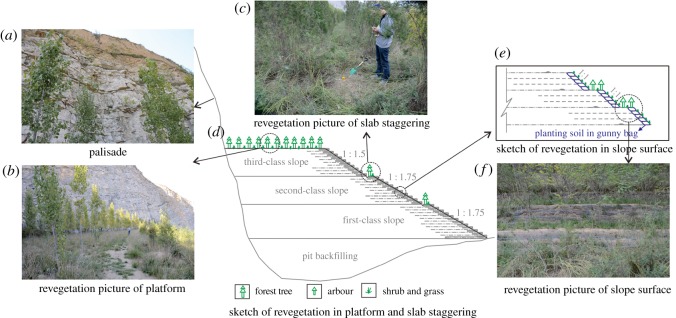
(*a*–*f*) Revegetation on the artificial slope.

The construction of the artificial slope provides conditions for revegetation. The artificial vegetation includes trees, shrubs and grass. In this case, considering the local climate and environment, specific species of trees, shrubs and grass were selected to guarantee their survival rate, focusing on the general species surrounding the mine area. The implementation of the revegetation measure is as follows:

(1) *Vegetation restoration*: on the platform and the staggered slope slabs, trees, including *Robinia pseudoacacia, Rhus typhina, Prunus Cerasifera* and *Calocedrus macrolepis*, were planted. The distance in each direction between the trees was 4 m, and the distance from the edge of the slope shoulder was 1 m. Among the trees, grass was planted, and the planting density was approximately 3000–4000 m^−2^ (figure 11*b*–*d*). On the slope surface, grass and shrubs, including *Agropyron cristatum, Medicago sativa, Caragana korshinskii* and *Artemisia desertorum*, were planted (figure 11*d*–*f*). In addition, the planting density is approximately 3000–4000 m^−2^.
(2) *Water storage*: in the work area, a reservoir is set up, collecting surface water and rainfall for pre-irrigation use. In this case, brick masonry is selected as the construction material, and the inside of the reservoir is lined with a waterproof polymer material.

### 3.2. Environment and ecosystem restoration

**Figure 12.**
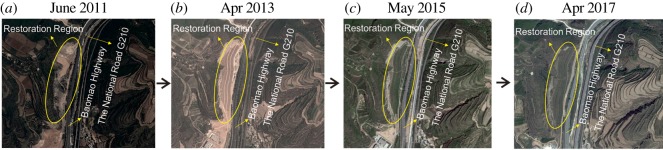
(*a*–*d*) Remote-sensing image of the quarry area in different periods.

**Figure 13.**
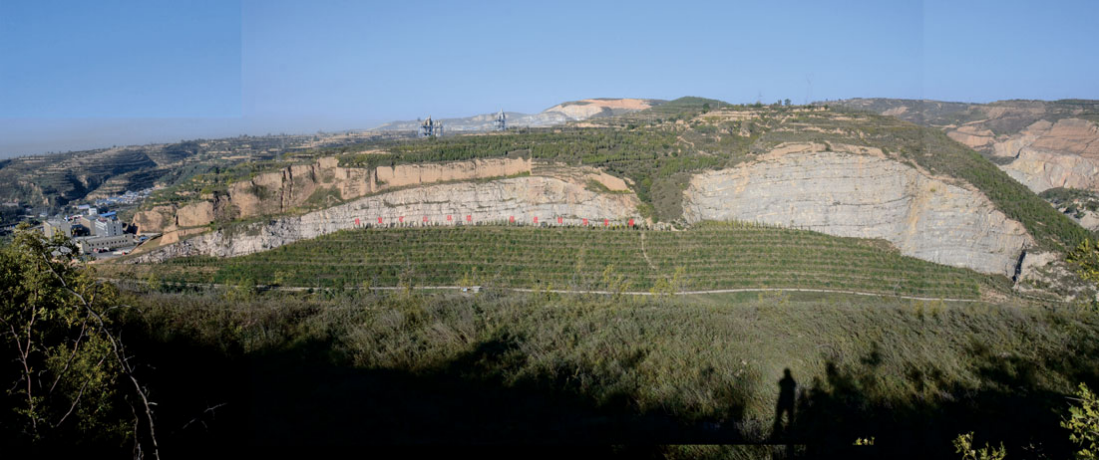
Restoration results picture.

The project officially started on 20 February 2012 and was completed on 30 June 2013. After comprehensive remediation, an area of 5.91 × 10^4^ m^2^ of land resources in the project area was reasonably recovered, and the restored total vegetation area reached 1.11 × 10^5^ m^2^ (figures 12 and 13).

In summary, through the implementation of the project, the local environmental problems including land degradation, land occupation, dust pollution and potential geological disasters were mitigated and eliminated, guaranteeing the safety of nearby engineering facilities and the security of personnel. Compared with the previous eco-environment, the artificial slope provided a favourable growth environment (figure 11). Moreover, human intervention accelerated the recovery process of the ecological environment. Through field investigation, the trees, shrubs or grass could mainly continue to live and grow well. Furthermore, the species are rich enough, which form a local ecology system that integrates into the surrounding environment (figures 12 and 13).

### 3.3. Artificial slope situation investigation

**Figure 14.**
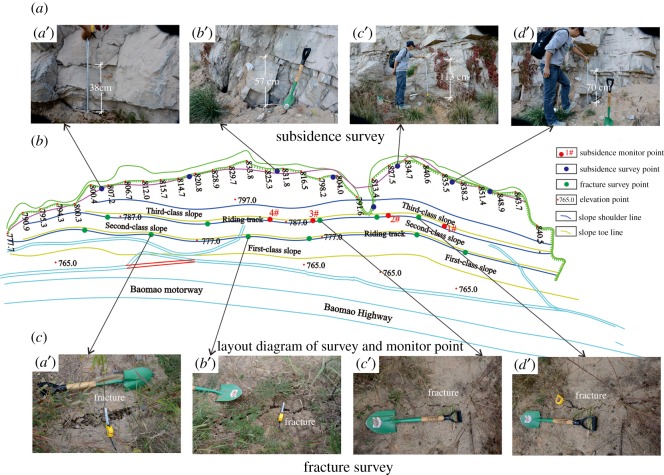
(*a*–*c*) Field investigation of the subsidence and cracking.

**Figure 15.**
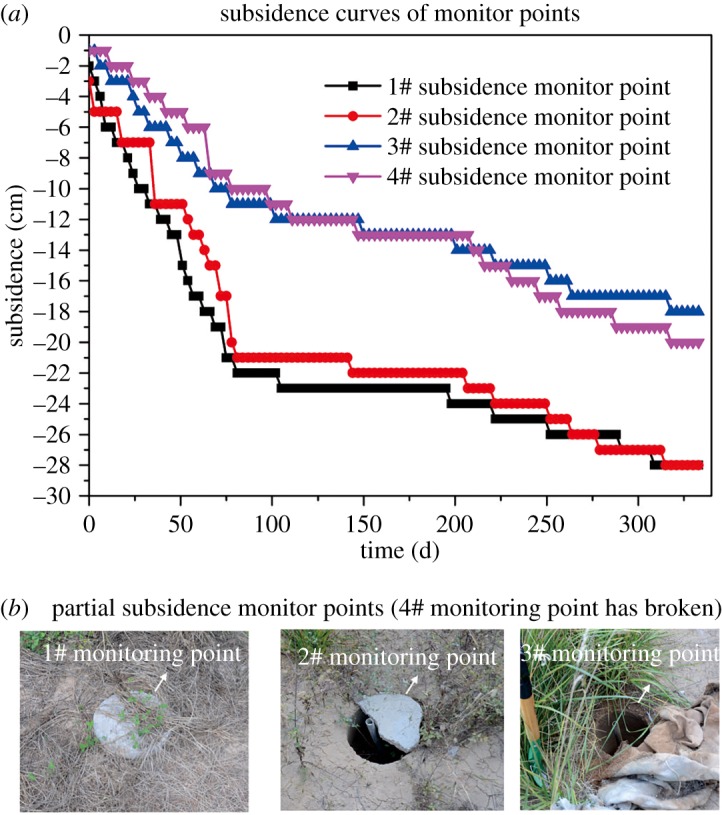
(*a*,*b*) Subsidence monitoring.

The field survey on the artificial slope was conducted in October 2015. For more than 2 years, the entire artificial filling slope produced a large consolidation subsidence. Furthermore, due to the change in thickness along the slope, the subsidence varies along the trend direction of the slope. The survey showed that, in the top seam position of the artificial slope and the rock palisade, the subsidence was approximately 30–100 cm. Four subsidence monitoring points were arranged in the second slope, 0.5 m away from the slope shoulder. Through continuous observation of the four subsidence monitoring points, the subsidence value of the second-class slope was recorded for nearly 1 year, beginning on 1 September 2013 (figure 14). Figure 15 shows that the final subsidence of the second-class slope is between 16 and 30 cm, and the subsidence period was mainly concentrated within 150 days after the completion of construction [38].

Moreover, due to the effect of the consolidation subsidence, tensile cracks with widths of 1–5 cm were produced at approximately 0.5 m from every class slope shoulder. In addition, these cracks were almost distributed along the whole trend direction of the slope. It is necessary to fill the tensile cracks with soil to prevent the infiltration of rainwater. However, during the whole investigation process, a shear outlet or potential shear outlet was not found on the slope. Therefore, the artificial slope had good stability.

## 4. Discussion

The environmental treatment method proposed in this paper is a novel and systematic idea and could be used to restore the ecosystem of an abandoned limestone quarry with deep open pits and steep palisades. Compared with previous studies [31,32,37,39], in this method, mixtures of rock debris, construction waste and loess were selected as the building materials, which overcame the deficiencies of high permeability of rock debris and construction waste and the collapsibility of loess. Moreover, the design of a multilevel slope guaranteed its own stability and facilitated revegetation.

With regard to the engineering case, the cost of the environmental restoration project in this abandoned limestone quarry was approximately 40 million Yuan. This project was conducted, solving the environmental problems in this quarry, restoring the local ecosystem and improving the local sustainable development. Through revegetation, a local ecosystem in the mine area was formed. As a result of manual selection, the species in the mine area are similar to but more abundant than those outside the vegetation cover. However, the development of vegetation and animal colonization in such mine areas will be investigated over a long time. In addition, this type of work is in process.

By combining the previous numerical simulation with the field investigation, the slope was found to have good stability. In general, vegetation is advantageous for slope stability, due to the anchorage effect of deep roots of woody plants on the slope, the reinforcement effect of shallow roots of herbaceous plants on the slope and the slope erosion prevention that the vegetation cover offers [40,41]. In addition, the field investigation showed that slope surface erosion clearly diminished. However, the study of the effect of vegetation on slope stability is complex. This topic will be paid close attention in the future work.

## 5. Conclusion

Considering the environmental problems and the availability of reusable resources, a novel method is proposed to restore the environment and ecosystem of abandoned limestone quarries. This method can effectively solve a series of problems, including eliminating or minimizing the potential geological hazards and dust pollution, and solving the land occupation and degradation problems. With the help of revegetation engineering, a previously damaged ecosystem could be restored. This method provides a good solution to treat the environmental damage caused by limestone exploitation, balancing the development between man and nature and providing a new idea for improving local sustainable development.

However, the problem of stability of the artificial slope and the subsidence problem are still difficult to solve. The artificial slope would produce a large subsidence and induce long and wide tensile cracks, which should be noticed during actual operation. The proposed method was successfully applied to an engineering case with steep palisades and deep pits. In analogous engineering projects, this method could be adjusted or improved according to the actual engineering situation to more precisely solve similar problems.

## Data accessibility

The datasets supporting this article have been uploaded to Dryad: (https://doi.org/10.5061/dryad.g48n010) [38].
